# Killing wolves to prevent predation on livestock may protect one farm but harm neighbors

**DOI:** 10.1371/journal.pone.0189729

**Published:** 2018-01-10

**Authors:** Francisco J. Santiago-Avila, Ari M. Cornman, Adrian Treves

**Affiliations:** 1 Carnivore Coexistence Lab, Nelson Institute for Environmental Studies, University of Wisconsin–Madison, Madison, Wisconsin, United States of America; 2 Department of Natural Resources, Little River Band of Ottawa Indians, Manistee, Michigan, United States of America; Universita degli Studi di Sassari, ITALY

## Abstract

Large carnivores, such as gray wolves, *Canis lupus*, are difficult to protect in mixed-use landscapes because some people perceive them as dangerous and because they sometimes threaten human property and safety. Governments may respond by killing carnivores in an effort to prevent repeated conflicts or threats, although the functional effectiveness of lethal methods has long been questioned. We evaluated two methods of government intervention following independent events of verified wolf predation on domestic animals (depredation) in the Upper Peninsula of Michigan, USA between 1998–2014, at three spatial scales. We evaluated two intervention methods using log-rank tests and conditional Cox recurrent event, gap time models based on retrospective analyses of the following quasi-experimental treatments: (1) selective killing of wolves by trapping near sites of verified depredation, and (2) advice to owners and haphazard use of non-lethal methods without wolf-killing. The government did not randomly assign treatments and used a pseudo-control (no removal of wolves was not a true control), but the federal permission to intervene lethally was granted and rescinded independent of events on the ground. Hazard ratios suggest lethal intervention was associated with an insignificant 27% lower risk of recurrence of events at trapping sites, but offset by an insignificant 22% increase in risk of recurrence at sites up to 5.42 km distant in the same year, compared to the non-lethal treatment. Our results do not support the hypothesis that Michigan’s use of lethal intervention after wolf depredations was effective for reducing the future risk of recurrence in the vicinities of trapping sites. Examining only the sites of intervention is incomplete because neighbors near trapping sites may suffer the recurrence of depredations. We propose two new hypotheses for perceived effectiveness of lethal methods: (a) killing predators may be perceived as effective because of the benefits to a small minority of farmers, and (b) if neighbors experience side-effects of lethal intervention such as displaced depredations, they may perceive the problem growing and then demand more lethal intervention rather than detecting problems spreading from the first trapping site. Ethical wildlife management guided by the “best scientific and commercial data available” would suggest suspending the standard method of trapping wolves in favor of non-lethal methods (livestock guarding dogs or fladry) that have been proven effective in preventing livestock losses in Michigan and elsewhere.

## Introduction

Large carnivores, such as gray wolves, *Canis lupus*, are difficult to protect in mixed-use landscapes because some people perceive them as dangerous and because they sometimes threaten human property and safety. Traditionally, governments kill wild animals in an effort to prevent threats to property and safety [1]. However, a recent summary of peer-reviewed studies that employed experimental or quasi-experimental tests of interventions against carnivore attacks on domestic animals in farms raised doubts about the functional effectiveness of lethal methods [2]. Namely, most tests of lethal methods showed no effect or counter-productive effects (higher livestock losses after intervention), and numerous tests contained biases or flaws that preclude reliable inference [2]. Two tests using quasi-experimental designs showed minimal, regional effect of various lethal methods [3] and a strong, local effect of government trapping and aerial shooting [4], respectively. But none provided the highest standard of evidence [2], which are random-assignment experimental tests of an intervention without bias in sampling treatment, measurement, or reporting [5, 6]. Higher standards of evidence were applied to tests of non-lethal methods generally, and two such tests applied the highest standards that also proved effective in preventing predation events on domestic animals (depredation). The two methods were fladry (a visual deterrent effective against wolves only, thus far) and livestock-guarding dogs [7, 8]. A recent controversy over killing wolves in the Northern Rocky Mountains (NRM) illustrates the difficulty of forming scientific consensus on the effectiveness of lethal methods for preventing depredations when standards of evidence are not consistent.

Two teams [4, 9] came to opposite conclusions when analyzing very similar data from the same region and similar period for the Northern Rocky Mountain wolf population. A deeper look suggests that inferences drawn from these quasi-experimental tests are weakened by uncontrolled variables (Box 1).

**Box 1**

One test included only wolf-killing by aerial gunning and several ground-based methods from 1989–2008 [4], whereas the other included all permitted wolf-killing, including public hunting, from 1987–2012 [9]. The latter of these two analyses found that killing more wolves was followed by more livestock losses the following year, using a negative binomial regression model controlling for multiple variables [9]. However, that test did not account adequately for the time series underlying several variables that increased over time. For example, over time the wolf population increased in size and also spread geographically, thereby exposing more farm animals to depredations. Because the amount of wolf-killing increased over time as (a) recolonizing wolves left the protection of a national park and wild areas, and (b) policy changes introduced wolf-hunting in addition to killing by government agents [4, 10, 11], we should expect the predictors (wolf-killing, livestock exposed, and wolf distribution) to rise over time in parallel with the observed rise in domestic animal losses over time, which would make a statistically significant association spurious if the time trend were not accounted for properly. Another team conducted the same analysis with the same data while accounting for time series trends and statistical misspecifications, and results suggest killing wolves instead led to an increase in attacks on cattle in the same year and fewer attacks the following year, relative to no killing [12]. However, this analysis seems to have eliminated the possibility of an underlying effect of wolf population size and did not consider the geographic spread of wolves, an approach that remains to be validated [12]. Proper statistical control for exposure (encounters between wolves and domestic animals) might require a measure of geographic spread of wolves, not just wolf and domestic animal abundances regionally. The remedy would have required spatial information at scales below that of the region. The authors of the analysis of wolf-killing between 1989–2008 incorporated spatial information, yet did not extend spatial analyses sufficiently, and limited their data to a time period when only government wolf-killing was legally allowed [4]. They found a reduction in risk of recurrence subsequent to wolf-killing within a wolf pack territory. The reductions appeared significant and high in magnitude after an entire pack was killed, and appeared significant but lower in magnitude when only part of a pack was killed, compared with no removal [4]. The analysis was restricted to the affected wolf pack territory, despite the researchers’ own work documenting how partial removal of wolves could scatter survivors beyond their original pack range [11, 13]. Therefore, the analysis of risk of recurrence of depredations should have examined neighboring areas and even more distant consequences. The importance of examining livestock loss beyond the edges of wolf pack territories had been noted [14]. We examine the analysis of [4] in greater detail in the Discussion.

We tested the hypothesis that two treatments (lethal and non-lethal intervention) following verified depredations had different effects on the risk of a recurrence (occurrence of a subsequent depredation) at that site and at neighboring sites at two larger geographic scales. We tested that hypothesis because the common justification for lethal interventions worldwide is that eliminating problem individuals, or regional predator reductions, will delay or curtail future losses immediately, and for at least one year until wolves are replaced [15]. We retrospectively examined data collected by state and federal agents in the state of Michigan, USA, from 1998–2014, using methods similar to [4], with two main differences. The first difference was that we examined spatial scales beyond the site of the intervention, so we could detect spill-over effects up to a radius of 16.25 km from the site of the intervention (neighborhood of township scale; see Methods section below). The second difference was that we included 2 distinct interventions: lethal and non-lethal interventions (pseudo-control, see below). Our analysis was retrospective and treatments had not been assigned randomly, thus the highest standard one might achieve would be a silver-standard experiment [2]. With data on the history and locations of events and interventions, we were able to draw stronger inference than a simple comparison of means between interventions. But quasi-experimental tests might be confounded by the effect of time passing (before-and-after) as carnivores, livestock, and people respond to changing conditions and other aspects of the environment change independently.

We had to consider potential bias in treatment. Field agents apparently made subjective judgments about where to implement lethal intervention when that was permitted by the federal government (Table 1 & [16]). Therefore, we had to contend with a pseudo-control as follows: At times, the state agency opted not to kill wolves or opted to offer farmers non-lethal deterrents, and the state advised the complainant on protection of livestock. The latter intervention involved communications and possible deployment of non-lethal deterrents (see below) with unknown characteristics or consistency. We also considered potential measurement errors–that may have been systematic, not random errors–associated with unreported wolf-killing and unreported depredations, both of which occur in neighboring Wisconsin [2, 14], and are believed to occur in Michigan as well [17, 18].

**Table 1:** Periods for wolf-killing policy signals in WI and MI, derived from Refsnider [16], ESA sec. 4 10(a)(1)(A) and Humane Society of the U.S. et al. v. Jewell (U.S. District Court, D.C., 5 1:13-cv-00186-BAH Document 52, 2014).

Period start (mm/dd/yyyy)	Period end (mm/dd/yyyy)	Federal status	Culling**
4/15/1994	3/31/2003	Listed as endangered	not allowed
4/1/2003	1/30/2005	Down-listed to threatened	allowed
1/31/2005	3/31/2005	Relisted	not allowed
4/1/2005	9/13/2005	Sub-permit for culling issued	allowed
9/14/2005	4/23/2006	Sub-permit rescinded	not allowed
4/24/2006*	7/31/2006	Sub-permit for culling issued	allowed
8/1/2006	3/11/2007	Sub-permit rescinded	not allowed
3/12/2007	9/28/2008	Delisted	allowed
9/29/2008	5/3/2009	Relisted	not allowed
5/4/2009	6/30/2009	Delisted	allowed
7/1/2009	26/1/2012	Relisted	not allowed
1/27/2012	4/14/2012	Delisted	allowed

*States identical except sub-permit issuance on 6 May 2006 to Michigan instead of issuance on 24 April 2006 to Wisconsin [16].

**Killing a wolf that posed a threat to human safety was always allowed under ESA sec. 11(a)(3).

Selection bias–or the tendency to apply different interventions to different subjects or locations based on some anticipated outcome–can powerfully affect the results of experimental tests [5]. In short, we controlled for spatial variation by comparing an intervention site to itself, but we could not control for the intervenors’ subjective decisions. In the Discussion, we identify and discuss potential sources of bias in the dataset provided to us.

Because of the caveats above relating to the strength of inference we might draw from the uncontrolled ‘experiment’ conducted by the State of Michigan, we regard our conclusions as preliminary in the same way that other recent published studies should be considered, pending gold-standard experiments [4, 9, 12]. These studies offer new inferences and testable hypotheses about the effect of interventions, rather than conclusions about the functional effectiveness of the interventions *per se*.

## Materials and methods

### Data sources

**Fig 1:**
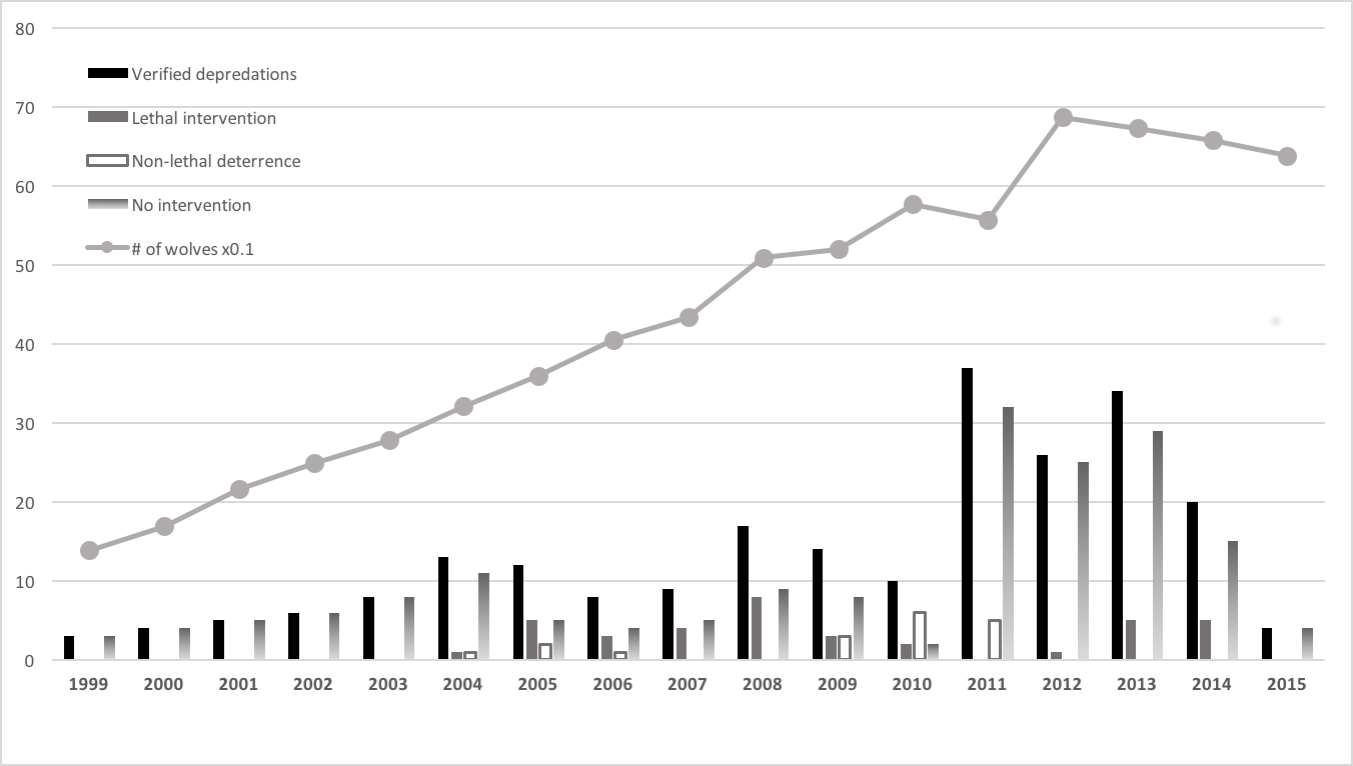
Annual Michigan wolf abundance, verified depredations and interventions. Michigan’s annual wolf abundance (divided by 10 to fit the same y-axis as other variables) and two treatments after verified depredations. The x-axis shows wolf-years, which span 15 April of year t-1 to 14 April of year t. Overall n = 230 depredations.

The State of Michigan continuously monitored complaints about wolves and annually monitored the wolves themselves, across the Upper Peninsula (42,610 km^2^). We used the federal government’s published reports for Michigan’s minimum, late-winter wolf population (https://www.fws.gov/midwest/wolf/aboutwolves/mi_wi_nos.htm), supplemented by Michigan data provided to the Little River Band of Ottawa Indians after their request through a federal Consent Decree. Michigan estimated wolf numbers by snow-track surveys, summer howling, and aerial telemetry of VHF radio-collared wolves primarily [19]. The exception was wolf-year 2012 when Michigan did not census its wolf population, so we interpolated the midpoint of the 2011 and 2013 estimates (Fig 1). Our study spanned wolf-years 1998–2015 (calendar-years 1998–2014); a wolf-year t was 15 April of year t-1 to 14 April of year t.

Michigan provided Wolf Activity Reports with 379 entries. The U.S. Department of Agriculture Wildlife Services (USDA) investigated many of these incidents since 1990 under state contract [20]. Hereafter, we refer to Michigan when referring to government responses to wolf-related complaints, whether by state or USDA field personnel. We discarded 149 entries that consisted of different categories of wolf encounters: observations, perceived threats to humans or domestic animals, or wolf interactions with hounds engaged in training or hunting, but which lacked verified depredations on a private property. Discarding perceived threats to humans should prevent the introduction of some biases, because the Wolf Activity Report entries suggested that one complainant’s ‘threat’ was another’s ‘encounter’ that did not result in official complaint, investigation or intervention. Considering the potential biasing effects of perceived threats that did not lead to a complaint (false negatives), perceived threats that were simply observations (false positives), and a complete lack of any such reports before 2002, we felt more secure setting aside all entries lacking depredations and ensuing verification. Also, wolf interactions with hounds occur under very different circumstances than depredations in our region [21–24]. In sum, we retained 230 complaints for screening as described below.

We screened complaints for verification and independence between depredations. During the study period, Michigan verified 499 livestock or farm animals injured or killed by wolves in 230 complaints. Depredations were classified as independent if they occurred on a different date.

Michigan responded in several ways to predation: communication only, provision of non-lethal deterrents, or lethal intervention. Lethal intervention consisted of live-trapping on or near the complainant’s property for several days to weeks after a depredation, and if successful, the state shot one or more wolves caught alive in leg-hold traps (n = 98 wolves killed overall, with lethal interventions following depredations in 37 occasions, and resulting in the deaths of 56 wolves in 32 interventions and 0 wolves killed in 5 occasions); in a few cases landowners shot wolves after receiving state permits. We omitted 32 cases in which wolves were killed but were not involved in depredations; only two of which occurred in the same townships (geopolitical mapping area of 36 miles^2^ or 92.16 km^2^) as lethal intervention during our study. We did not include the public hunting season at the end of 2013 because those removals were not targeted at known complaint sites [25]. Non-lethal deterrence was used primarily when no losses occurred in the Wolf Activity Reports, so most such interventions were excluded by our screening criteria above.

We refer to any intervention that did not lead to wolves dying as non-lethal, which implies only that no wolves were killed, but related actions may have entailed a range of communications with the complainant and other responses, including the provision of non-lethal deterrents in some cases. All interventions included communications with complainants but we had no data to determine if such communications differed between lethal intervention and non-lethal. Non-lethal deterrents included one or more of the following: cracker shells, hazing kits, live-traps, lights, or fencing with various materials, including fladry (a loose flagging hung at regular intervals on fence-lines [26]). We also classified live-trapping (i.e., attempted lethal interventions) that resulted in no wolves killed (n = 5) as ‘non-lethal’. Differences in non-lethal methods implemented at different sites could be attributed to costs, judgments by state agents about effectiveness in a given situation, willingness of livestock owners to deploy certain techniques, or other undocumented factors. Because of the small sample of occasions when non-lethal deterrents were deployed after depredations (n = 18), we pooled all interventions that did not lead to wolf-killing as non-lethal, due to insufficient information on whether the deterrents were actually implemented by the farmer.

A true control would have enacted all the same procedures and time spent on the complainant’s property without killing wolves, or installing any non-lethal infrastructure. Therefore, we refer to our non-lethal intervention classification as a pseudo-control because it may have included different communications or a judgment by a state agent that lethal intervention was not likely to succeed. However, given that the federal permit for the state to use lethal control was issued and rescinded several times without regard to events on the ground (Table 1), we infer that the two treatments we analyzed were largely selected because of the broader governmental timelines rather than the events at a particular property. Independent decisions about the availability of lethal intervention would reduce the risk of treatment bias [2]. Regardless, this study represents a silver-standard experiment with possible treatment biases that must be considered preliminary and examined carefully (see Discussion).

With the preceding criteria, our primary sample of 230 depredations (or depredation events, by which we mean a verified, independent wolf depredation incident in the Wolf Activity Report) consisted of 32 depredations followed by lethal intervention, and 198 followed by non-lethal intervention.

### Analyses

We used geopolitical sections (regular units of 1 mile^2^ or 2.56 km^2^) as the smallest mapping units, following [27]. Sections can be read from commercially available road atlases. Sometimes more precise locations were also provided, but inspection revealed that many of these were simply the latitude and longitude of the center of the section. Virtually every livestock pasture lay within the borders of a single section. All livestock pastures were on private property of much less than 1 section in area (average farm size was 0.3 miles^2^ or 0.68 km^2^ in the Upper Peninsula [28]). The state did not record ownership of pastures or the tenure status of complainants. All depredation events are presented in **S1 Data File** with certain personal details, property information, and precise locations redacted for privacy.

We determined the sequence of depredation events by reference to the date of the complaint on the Wolf Activity Reports. We calculated the delay to recurrence as the interval in days to the next event in the same vicinity (2.56 km^2^ section or larger geographic unit, see below). If there were no subsequent events in the vicinity that calendar year, we censored that observation of delay to recurrence at 31 December of the same year. Virtually all depredations occurred in the warmer months [20], with most events occurring in the period March-October (90%) and only 3% occurring in November or December, echoing results from Edge et al. [18]. Livestock in the Upper Peninsula are kept within enclosed pastures year-round, usually in small farms, and thus equally available to wolves throughout the year [18, 29]. Therefore, our decision to measure and censor the delay to recurrence within the calendar year provided at least 60 days to detect an effect in 97% of events (recurrence at section scales occurred within a median of 13 days if it occurred the same year). Had we extended the time horizon as in [4], we saw a risk of conflating the recurrence of depredation events by later wolves with the treatment applied to prior wolves.

**Fig 2:**
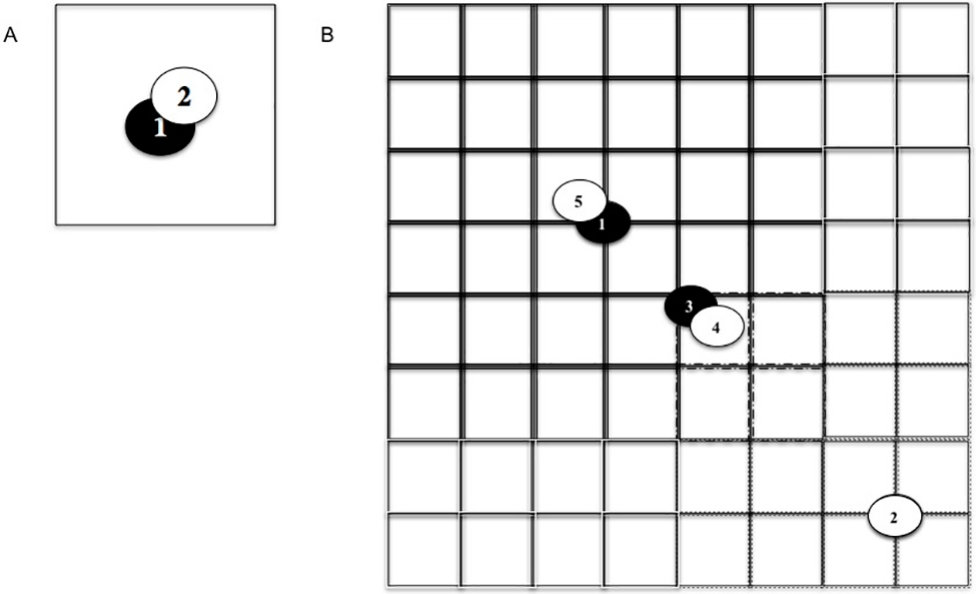
Measuring recurrence between depredation events at multiple spatial scales. Each small rectangle is a section (1 mile^2^). Each oval is a single event of verified depredation. A 1 indicates the first of such events in its vicinity and year, and higher numbers are subsequent events in chronological order of occurrence in the same year. The intervention is shown with colored ovals: lethal (black), non-lethal intervention (open); and events within the same section are depicted as overlapping each other partially (1 and 2 in A; 1 and 5 or 3 and 4 in B). **A**: Smallest scale of analysis where the vicinity is limited to the section. Datum 1 stratum 1 measures the number of days between events 1 and 2 with lethal intervention. Because there is no event 3 within the vicinity, datum 1 stratum 2 measures the number of days between event 2 and the end of the calendar year but switches to non-lethal intervention (open oval). **B**: Medium-scale of analysis where rectangles are sections in a township (36 miles^2^ centered on event 1). Solid black grid lines indicate buffer around event 1; dotted gray lines indicate buffer around event 2; black dot-dashed lines indicate overlap between buffers. Because event 1 and event 2 are not in the same township-sized buffer, they generate datum 1 and datum 2 with lethal intervention and non-lethal intervention, respectively. Datum 1 stratum 1 measures the number of days between event 1 and event 3. Although event 3 is also within the buffer of event 2 (within black dot-dashed lines), it was assigned to event 1 because it was nearest by Euclidean distance. We did not measure the number of days between events 2 and 3 because event 3 was already used to create datum 1 stratum 1; in this way, we avoided double-counting events. Next, events 3 and 4 are collapsed (treated as a single event) because they occurred in the same section *sequentially*. Because event 3 was followed by lethal intervention (black oval), the resulting single collapsed event was classified as lethal intervention. We then measure datum 1 stratum 2 as the number of days between event 4 and 5, remembering that the collapsed event is classified as lethal even though 4 is followed by non-lethal intervention (any collapsed set of events with a lethal intervention event among them is assigned to the lethal intervention set). Finally, datum 1 stratum 3 is measured by the number of days between event 5 and the end of the calendar year and assigned to non-lethal intervention. If event 2 had zero other events in its township area (not shown), then datum 2 stratum 1 would be measured to the end of the calendar year. A similar process was followed for the largest spatial scale of neighborhood of townships (320 miles^2^).

We also examined if depredations recurred at two larger spatial scales. At the intermediate scale of townships (36 miles^2^ or 92.16 km^2^), the area used for measuring recurrence approximated half the core area of an average wolf pack territory [30]. At our largest spatial scale, the neighborhood of townships (320 miles^2^ or 829.44 km^2^) was equivalent to 9 contiguous townships centered on a depredation event and >4 times the average core area of a wolf pack territory [30]. For analyses of risk of recurrence at the township and neighborhood scales, we replaced the fixed geopolitical unit with a square buffer of the same area centered on each depredation event (Fig 2). We detected no difference in the sequence of depredation events for particular areas when using a circular buffer, possibly due to the coordinates for depredation incidents obtained from the Wolf Activity Reports frequently placing the incident in the center of a section, which both buffer shapes contained. The square buffer was preferred based on its consistency with the underlying Public Land Survey System (USGS, https://nationalmap.gov/small_scale/a_plss.html) layer containing the spatial subdivisions we based our three spatial scales on. We measured delay to recurrence in that buffer, repeating the process for subsequent depredation events using each event’s buffer (i.e., a moving window). The process of assigning depredation events at scales larger than the section (Fig 2) was designed to avoid pseudo-replication (once the effect of a pair of events was measured at a lower scale, that estimate of delay to recurrence was never used again at larger scales).

The use of three spatial scales allowed us to detect depredation recurrence beyond the original sites (spill-over effects) following interventions. Our process for collapsing depredation events (Fig 2) produced a conservative assessment of spill-over effects because we eliminated pseudo-replication of estimates of risk of recurrence across scales. The disadvantage of our approach was declining sample sizes that reduced the power of the tests at larger scales and thereby potentially increased Type II error.

### Statistical tests

**Fig 3:**
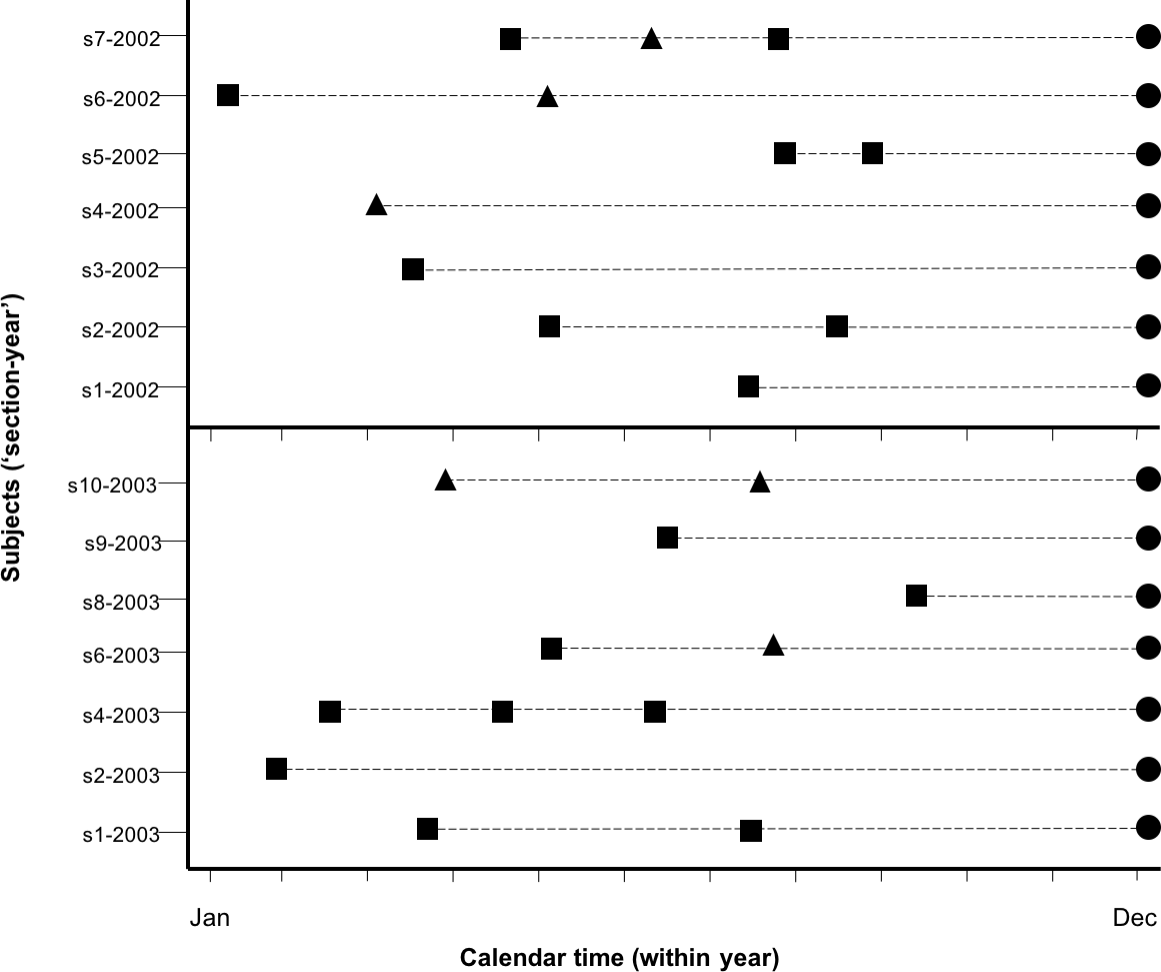
Transforming depredation records to a survival analysis format. We present lethal interventions (triangles) and non-lethal interventions (squares) connected by a dashed line that measures the delay to recurrence or censorship (circles). We illustrate using data from two subsequent years. Subjects are identified as combinations of vicinity (section, township or neighborhood) and year (i.e.: section s1-2002) on the y-axis. The first figure for each subject represents when the first depredation event in that year occurred, which is the date follow-up started for that ‘section-year’. Each subject then follows a chronology of subsequent depredation events through the year, treated with either intervention. Stratum 1 considers the initial intervention implemented and the delay to recurrence to the next depredation event, or censoring if no other events occurred (i.e.: first figure to second figure in dashed line for each subject). Stratum 2 considers the next sequence of depredation events (i.e.: delay from second figure to third figure). Due to our construction of subjects, a particular section (sections 1, 2, 4 and 6, for example) can appear in multiple years, represented with a different ‘section-year’ combination (for example, s1-2002 and s1-2003).

We measured delay to recurrence in days between each pair of successive depredation events as in Figs 2 and 3, and produced survival functions for each treatment following Hosmer, Lemenshow & May [31]. A survival function describes the probability of observing a time interval between two depredation events, *T*, greater than some stated value *t*, S(*t*) = P(T>*t*), where *t* is days. Thus, survival functions provide, for every time *t*, the probability of ‘surviving’ (in this case, not experiencing a depredation event) up to that time, and describe these probability distributions (survival distributions). Survival analysis comprises a set of statistical methods used to quantify and test survival function differences between treatment groups of subjects [32].

At the smallest spatial scale, we defined our subjects as the sections in which depredations occurred. Thus, sections are analogous in biomedical research to the patient receiving treatments. In this case, the section receives lethal or non-lethal treatment of wolves. Note that this differs from prior research that defined wolf pack territories as the subjects [4].

Subjects enter the analysis after the initial depredation event, and remain in the analysis until December 31^st^ of that year; hence, our subjects arise from a particular vicinity (i.e., section, township or neighborhood) in a particular calendar-year (1998 to 2014) (Fig 3). Depredation events, along with their respective treatments and measures of recurrence were organized into strata based on their order of occurrence for each subject (Fig 2). Each year a new set of strata was created, starting with stratum 1 again. The end of each calendar year represented a ‘reset’ point after which we assumed independence of subjects because both wolves and livestock are mostly removed from each other’s reach until the next grazing period. Based on this classification of subjects and strata, we clustered our analysis on a unique identifier reflecting a particular vicinity-year combination, e.g., ID_TRS_Yr [33]. This approach accounts for potential spatial and temporal auto-correlation among strata within subjects, e.g., all depredation events for the same subject experienced during a particular year are assumed correlated. It also avoids pseudo-replication of observed depredation events from the same subject as if they were independent of other depredation events in that same year, e.g., ID_TRS_2000’s stratum 1 and stratum 2 observations are correctly identified as belonging to the same subject, rather than belonging to two different subjects (pseudo-replication). In the Discussion, we examine potential pseudo-replication concerns in our dataset and in prior approaches.

We employed general and stratified log-rank tests (Chi-squared statistic) to compare the survival distributions for delay to recurrence in both treatments. We then used a conditional Cox recurrent event, gap time model [31] to compare the associations between treatments and risk of recurrence. The Cox model allowed us to estimate hazard ratios (HR) for relative risk of recurrence between treatments by characterizing how the hazard function (H) changed as a function of survival time and subject covariates; S(*t*) = e^-H(*t*,*x*,*ß*)^, where *t* is study time (the period of observation or follow-up period after inclusion in study until end of the calendar year), *x* is a covariate we describe below, and *ß* is the parameter estimate of *x*.

The *stratified* conditional Cox model accounts for risk of recurrence for the i^th^ depredation event being influenced by the occurrence of a previous (i-1)^th^ depredation event and the treatment following it, so that each subject is included in the risk set (the number of subjects experiencing a depredation event) for the i^th^ depredation event only if it experienced the (i-1)^th^ depredation event. For example, in our section-scale analysis, 31 subjects experienced a first recurrent depredation event, whereas 120 did not experience any recurrence (Stratum 1, Tables B & C in S1 File).

The stratified Cox model considers only those subjects experiencing that first recurrent depredation event in the second stratum (Stratum 2, n = 31; Table A in S1 File), repeating the process for subsequent strata until end of the calendar year. The stratified Cox model allowed us to estimate general treatment effects while accounting for event order and the treatment applied to the previous event.

We ran univariate and multivariate conditional Cox models at each spatial scale. Univariate models included only our response variable (delay to recurrence) comparing our two treatments, whereas multivariate models incorporated calendar year. Including calendar year was essential because the gray wolf was down-listed to threatened in Michigan on April 1, 2003, and subsequently went through 12 or more reclassifications and permit issuances that precluded or allowed wolf-killing by the state ([34], and Table 1]) as the protection afforded wolves was reduced or increased.

Given that treatment effects could change over time as wolves, livestock, people, and ecosystems might change with environmental conditions, we also ran multivariate models incorporating a time-varying covariate (tvc) for treatments [31]. Our tvc consists of an interaction of treatment with study time. The use of a tvc is strongly recommended for evaluating and handling non-proportional hazards (PH), given PH is an underlying assumption of survival modelling [31]. A non-proportional hazard occurs when the treatment effect changes over time (instead of remaining constant) relative to the pseudo-control, so that the hazard ratio for the treatment changes over time. Hence, if the parameter estimate for the tvc were found to be significant, the conditional Cox model with tvc would be more robust and reliable than without the tvc because it corrected for non-proportional hazards in our treatments. When the tvc is not significant, its inclusion in the model is not warranted.

Authorities on stratified Cox models also express concerns about strong inference depending on the risk set per stratum [31, 35]. The latter authors did not settle on a particular number observations per treatment per stratum; however, the Cox models depend on a measure of variability within-strata to detect deviations from chance differences between treatments, therefore we excluded strata with <10 depredation events or which lacked events for both treatments. This conservative step left us with 3 strata at the section scale, 1 stratum at the township scale, and 2 strata at the neighborhood scales (S1 File). Thus, our final sample at the section scale consisted of 151 subjects (independent section-years) with 199 depredation events, including 56 recurrent depredation events; the final sample at the township scale consisted of 125 subjects with 125 depredation events, including 24 recurrent depredation events; and the final sample at the neighborhood scale consists of 106 subjects with 125 depredation events, including 25 recurrent depredation events (S1 File).

We assessed the robustness of models to within-subject correlation by running a variant of a random-effects approach called frailty models ([35]; S2 File). If high-risk and low-risk farms exist due to factors extrinsic to treatments, years, or the tvc, then subject identity should inform gap time models [17, 36]. Frailty models assess the goodness of fit of the treatment variable by including random effects of subject identity [35], which is considered useful when recurrence time might be influenced by unmeasured factors [31, 37].

We also built models with subsets of the data to evaluate potential confounding effects and robustness of the primary models described above. We built a model with data ‘post-2003’, after lethal management was episodically permitted, and by reclassifying lethal management with zero wolves killed as ‘lethal’ because the infrastructure and attendant human influences would be the same whenever traps were laid regardless if wolves were live-trapped and killed. We refer to the latter condition as ‘traps placed’. We present alternative models in supporting information (S2–S4 Files).

Finally, we used Spearman rank correlations (r_s_) to correlate delay to recurrence with number of wolves killed for lethal treatments only and for ‘traps placed’. We conducted all analyses in Stata 14 (StataCorp, College Station, TX, 2015; protocol DOI: 10.17504/protocols.io.j2rcqd6).

## Results

Between 1998 and May 2014 there were 199 depredations in Michigan with as many management interventions. Of the 199, 31 resulted in lethal intervention (16%) and 168 resulted in non-lethal intervention (84%) (Fig 1).

### Section scale

Log rank tests could not distinguish the survival functions between treatments (df = 1, general survival functions test: χ^2^ = 0.27, P = 0.604; stratified [by order of depredation events for subjects] test: χ^2^ = 0.48, P = 0.488; Table 2). All univariate (treatment only) and multivariate (treatment and calendar-year) Cox models suggest that lethal intervention was associated with a non-significant reduction in risk of recurrence when compared to non-lethal intervention (Table 3). The section-scale models including a time-varying covariate (tvc) were not significant, so the PH assumption was not violated (tvc P>0.05). The multivariate model including treatment and year suggests lethal intervention only weakly reduced risk of recurrence (slowing recurrence) by 27%, but that was not a statistically significant difference (HR = 0.73, P = 0.326; Table 3). This model also revealed an increasing risk of recurrence (hastening recurrence) by 9% each calendar-year (HR = 1.09, P = 0.022). Lethal intervention was not significantly different from non-lethal intervention in our frailty model (HR = 0.48, P = 0.158; Table A in S2 File), with the model suggesting significant frailty (omitted or unobserved covariates) remaining in the model (P = 0.006). For those depredation events followed by lethal intervention, we found no correlation between delay to recurrence and the number of wolves killed (Spearman’s rho = 0.107, P = 0.559, Table 4; ‘traps placed’: Spearman’s rho = 0.076, P = 0.657; Table C in S3 File).

**Table 2:** General and stratified log-rank (χ^2^) tests examining difference between treatments’ (lethal and non-lethal) survival distributions (measuring risk of recurrence) after wolf depredations, for all spatial scales.

	Spatial scale of analysis		
	Section	Township	Neighborhood
***SUBJECTS AND 'FAILURES'***			
**TOTAL DEPREDATION EVENTS**	199	125	125
Failures (recurrent events)	56	24	25
***SURVIVAL FUNCTIONS***			
**Log rank test (χ2)**	0.27	1.44	0.08
p-val	0.603	0.23	0.772
**Stratified Log-rank test (χ2)**	0.48	-	0.28
p-val	0.488	-	0.593

**Table 3:** Main results of Cox models measuring risk of recurrence between treatments (lethal and non-lethal) implemented after wolf depredations, for all spatial scales.

	Spatial scale of analysis					
	Section		Township		Neighborhood	
*PROPORTIONAL HAZARD MODELS*	*Interv*	*Interv & year*	*Interv*	*Interv & year*	*Interv*	*Interv & year*
**Standard cox (stratified)**						
*Intervention HR (SD)*	0.77 (0.22)	0.73 (0.23)	0.48 (0.308)	0.46 (0.29)	0.80 (0.340)	0.72 (0.34)
p-val	0.36	0.326	0.255	0.224	0.644	0.486
*year HR (SD)*	-	1.09 (0.04)*	-	1.05 (0.05)	-	1.14 (0.07)*
p-val	-	0.022	-	0.28	-	0.024
**Standard cox with tvc (stratified)**						
*Intervention HR (SD)*	0.48 (0.21)*	0.46 (0.21)	1.87 (1.47)	1.78 (1.38)	0.84 (0.62)	0.80 (0.63)
p-val	0.099	0.091	0.425	0.458	0.818	0.778
*tvc(Intervention) HR (SD)*	1.01 (0.01)*	1.01 (0.01)	0.97 (0.01)**	0.97 (0.01)**	0.99 (0.01)	1.00 (0.01)
p-val	0.057	0.068	0.001	0.001	0.928	0.852
*year HR (SD)*	-	1.09 (0.04)*	-	1.05 (0.05)	-	1.14 (0.07)*
p-val	-	0.023	-	0.281	-	0.023

Significance

* if p-val < .05

** if < .01.

**Table 4:** Spearman correlation between delay to recurrence and number of wolves killed after depredation events followed by lethal intervention (wolves killed ≥ 0), for all spatial scales.

	Section	Township	Neighborhood
Spearman's rho	0.107	0.212	0.295
p-val	0.5591	0.2994	0.1354

### Township scale

Our dataset consisted of 125 depredations, 26 followed by lethal intervention (21%) and 99 followed by non-lethal intervention (79%). Log rank tests could not distinguish the survival functions between treatments (df = 1, general test: χ^2^ = 1.44, *P* = 0.23; Table 2). Likewise, all Cox models revealed no significant differences between treatments (Table 3). The township-scale models including a tvc were significant, suggesting the PH assumption was violated (tvc P<0.05). Hence, we focus our analysis on the model including the tvc. Lethal intervention increased risk (hastening recurrence) by 22%, but this was not statistically significant (treatment HR = 1.78, P = 0.458). However, our tvc, which accounts for non-proportional hazards, hints at a minimal (3%) reduction in risk over follow-up time (tvc HR = 0.97, P = 0.001). Calendar-year was not significant (HR = 1.05, P = 0.281). Differences between treatments were not significant in our frailty model (HR = 0.45, P = 0.242; Table A in S2 File). For those events followed by lethal intervention, we found no correlation between delay to recurrence and the number of wolves killed (Spearman’s rho = 0.212, P = 0.299, Table 4; ‘traps placed’: Spearman’s rho = 0.233, P = 0.224; Table C in S3 File).

### Neighborhood scale

Our dataset consisted of 125 depredations, 26 followed by lethal intervention (21%) and 99 followed by non-lethal intervention (79%). Again, log rank tests could not distinguish survival functions between treatments (general test: χ^2^ = 0.08, *P* = 0.772; stratified test: χ^2^ = 0.28, *P* = 0.594). Similarly, all Cox models revealed no differences between treatments (Table 2). The neighborhood-scale models including a tvc were not significant, so the PH assumption was not violated (tvc P>0.05). Lethal intervention only weakly reduced the risk of recurrence (slowing recurrence) by 28% but this difference was not significant (treatment HR = 0.72, P = 0.486; Table 3). We found a statistically significant increase in risk of recurrence (hastening recurrence) of 14% every calendar-year (HR = 1.14, P = 0.024). The frailty model showed no significant differences between treatments (HR = 0.80, P = 0.67; Table A in S2 File).

For those events followed by lethal intervention, we found no evidence of a correlation between time to recurrence and the number of wolves killed (Spearman’s rho = 0.295, P = 0.135, Table 4; ‘traps placed’: Spearman’s rho = 0.161, P = 0.395; Table C in S3 File).

For all spatial scales, all effects of treatment remained consistent for the ‘traps placed’ condition, when limiting the data to post-2003 depredation events, ‘skip-a-year’ dataset and when removing a special case (S2–S4 Files).

## Discussion

We retrospectively evaluated whether lethal interventions by the State of Michigan in response to wolf predation on domestic animals (depredations) between 1998–2014 resulted in lower risk of recurrence of depredations than if no wolves were killed. We found the delay to recurrence of depredations was unrelated to the number of wolves killed at all spatial scales. We found lethal management did not significantly shorten or lengthen the interval to the next depredation relative to non-lethal interventions. A small, statistically insignificant reduction in the risk of depredation at the section level was offset by a similar and also statistically insignificant increase in the risk of depredation at the township scale, which is about half the size of a wolf pack territory, and then a similar decrease in risk at the scale of neighborhoods of townships, which are four times larger than the average wolf pack territory [30]. None of these differences were statistically significant using a battery of tests.

Our methods or alternative models accounted for potential violations of the proportional hazards assumption, unlike a prior study of wolves in the Northern Rocky Mountains (see below); accounted for within-subject correlation; were unaffected when we restricted analysis to the period after 2003 when lethal interventions first became legal; and accounted for a change in definition of lethal methods to include the installation of lethal methods that did not kill any wolves (S3 File). There is evidence for the effect of lethal intervention changing slightly over the course of a single calendar year at the township scale, through a minimal reduction in risk over follow-up time. We also detected variation between individual farms in their time to recurrence of depredations. Given the apparent, net ineffectiveness of lethal intervention and the uncertainty about potential biases in a retrospective analysis of sparsely documented government interventions, we recommend ethical, gold-standard, random-assignment experiments be used before further lethal management is authorized to prevent depredations.

Overall, our analysis suggests that any potential beneficial effects of lethal interventions locally would be offset by detrimental effects for neighboring farms in the same township. If the small, local improvements were considered biologically, ethically, or economically important to one farm, then one would also have to admit the associated costs to neighboring farms and the biological, ethical and economic importance to that farm. Therefore, given the evidence available, we cannot conclude that lethal management had the desired effect of preventing future livestock losses.

Over the 17 years of our study, the risk of depredation increased by 9 and 14% per year at the section and neighborhood (smallest and largest) scales, respectively, in our main dataset. However, this effect of year is insignificant in our post-2003 dataset (S4 File). In addition to changes in wolf densities locally that may have occurred, there may also have been changes in proportion of pasture, prey density, land cover, farm size, road density, among other variables that predict depredations at local scales [17, 38]. Also, prior work indicated smaller packs were more often implicated in livestock depredations than larger packs [23]. Therefore, the notion that higher densities of wolves locally will result in more depredations is not well supported, as opposed to the idea that a recolonizing population encounters more livestock as a result of recolonizing more and more of their historic range over time.

We present our results guardedly rather than as a definitive conclusion about effectiveness because of insurmountable uncertainties about the government data. Retrospective analyses to evaluate the effectiveness of interventions to prevent predation on livestock are fraught with uncertainty because of various biases or challenges presented by field conditions [2]. For example, treatments were not assigned randomly and changing conditions over time locally were not documented. The unintentional error may have been random but we are unable to rule out systematic error (bias), whether intentional or unintentional. The government dataset we analyzed had undocumented variability in data collection and intervention, including possible systematic selection bias affecting which areas received which interventions.

Selection (or enrollment) bias would arise if subjects entered the study under varying conditions that affected outcomes. All sections containing farms (subjects) entered our study because of a verified depredation, but subjects entered at different times and some farm owners might have responded to depredations in undocumented ways including poaching wolves. Likewise, attrition bias would arise if subjects left the study for reasons that were not random with respect to their outcomes. This would occur systematically if a subset of the interventions led farmers not to complain in the future despite facing depredations, or to take matters into their own hands, as above. Compensation was offered throughout the study as well as state-financed non-lethal deterrence when lethal intervention was unavailable, so attrition by withholding complaints seems unlikely to have been frequent or widespread. However, we would guess that non-intervention might be construed as unhelpful by complainants, leading some of them to intervene independently. We consider unreported wolf-killings to be a more pronounced confounding variable after 2003, when state lethal management was allowed (Table 1), substantiated by a recent inference that allowing state killing of wolves seems to have potentially increased poaching of Michigan and Wisconsin wolves [34]. By definition, poaching can only confound tests of non-lethal deterrence because poaching following lethal intervention would only increase the number of wolves killed (undetectably in our context), but not change the nature of that lethal intervention. We do not see how poaching could confound the apparent reversal of effects of lethal control across our three geographic scales of analysis.

Furthermore, treatment bias would arise if methods of intervention were not standardized. Treatment bias certainly arose among non-lethal deterrents because different complainants received different types of non-lethal methods and we do not know if they maintained or installed the methods appropriately or identically. Non-lethal deterrents were presumably negotiated with complainants and therefore most prone to treatment bias that would confound our results. However, only 8% of our eventual sample received non-lethal deterrents. Moreover, we have no data on other deterrents or precautions unilaterally implemented by complainants. Lethal interventions were more uniform in method [20] but we did not receive precise, detailed information on implementation (number of trap-nights, exact locations, etc.). Moreover, if lethal interventions were spatially segregated from other types of interventions, then selection bias might have applied systematically because farms perceived to be higher-risk might have received lethal interventions preferentially and also be expected to have recurrent depredations. This might have resulted in significant, between-subject variability. Such a bias would not explain the spill-over effect we detected. Intermittent authority for lethal intervention led to the same spatial units receiving all types of intervention (**S1 Data File**). Given that authority for the state to kill wolves after verified depredations was granted or withheld by federal decisions unrelated to area attributes or recent depredation complaints and in several years of the study even high-risk areas received no interventions [16], it seems unlikely that lethal control authority for Michigan coincided with risky years. Therefore, any treatment bias (intervening lethally at sites that were inherently more likely to have recurrence of depredation) would have to occur at the spatiotemporal scale of individual farms within years. We addressed within-subject variability using a frailty model (S2 File), which revealed the presence of confounding effects at the section level, but the treatment effect remained statistically insignificant.

Finally, wolf abundance was unlikely to confound our tests because the number of wolves within our spatial units was unlikely to change substantially from one incident to the next within a small area within one year.

In sum, we find ample reason to expect confounding variables would weaken inference from a retrospective, quasi-experimental test of interventions to prevent livestock loss. Our attempts to detect and screen for biases were necessarily imperfect because we could not assign treatments randomly nor could we retrospectively assess if interventions were assigned haphazardly or subjectively. Our analyses controlled for variation in risk due to time and inter-farm differences using tvc and frailty models (S2 File), but could not ultimately control for transient changes in risk associated with wolves, people, or other wildlife. Moreover, we were not able to account for illegal wolf-killing that might have added to treatment bias affecting non-lethal interventions.

Nevertheless, there is value in the scientific examination of on-the-ground programs of predator management as they are actually carried out by the organizations that discharge them. Avoidance of selection, treatment or measurement biases would require enforcement of strict protocols that are rare worldwide [2, 39–41]. In addition to understanding how the strongest inference arises from gold-standard experiments without bias, wildlife managers have a responsibility to continually evaluate their particular actions and policies to ascertain if they are effective at accomplishing the goals set by the broadest society, and to remedy or terminate them if they are found to be ineffective, as evidence-based policy-making demands.

An example of a gold-standard design that might achieve strong inference would be random-assignment of treatment to different, large areas (e.g., 324 km^2^) with uniform treatments, in which measurement is unbiased by blinding or independent, third party monitors, and data analysis is conducted by independent, third-party analysts without financial conflicts of interest involving the government or livestock industry. However, such an experiment would have to address the ethical implications for both animals and people of removing wild animals, possibly exposing more livestock to spill-over effects, and the broad public interest in preserving both wildlife and livelihoods. A step in that direction, albeit imperfect, may be to temporarily relocate predators to captivity until the analysis period ended in each area.

If our results are supported by a gold-standard experiment, we propose a hypothesis for two long-standing phenomena about human perceptions of conflicts with predators and the perceived effectiveness of interventions. We observe that killing predators is widely perceived to be effective (e.g., in our region: [42, 43], yet afterwards real and perceived risks appear to increase [44]. The spill-over effect may be responsible. Our hypothesis builds on the idea first articulated by Haber [45] that killing wolves can trigger pack disruption which might lead to more livestock predation than done by intact packs. If our inference about spill-over effects is confirmed, then we hypothesize that the perceived effectiveness of lethal methods stems from a few livestock owners who report preventive benefits, while neighboring livestock owners report increasing losses because of the spill-over effect from the former farms. The adverse effects of killing wolves as a response to depredations might thereby be obscured by anecdotal accounts and misperceptions.

Our results appear to contradict those of the [4] in the Northern Rocky Mountains (NRM) for the period 1989–2012. Although [4] conducted similar survival analyses, they found lethal methods significantly reduced the risk of recurrence, and that killing an entire wolf pack was more effective than the killing of a subset of members of a pack. They reported only a marginal difference between partial pack removal and no removal if wolves were killed within the first 7 days following a depredation event and no difference if 14 days elapsed. Most lethal interventions in Michigan were probably partial pack removals (median wolves killed = 1, **S1 Data File**) so our results are consistent. However, other differences in results between their study and ours could be due to different sites and methods.

The analysis in [4] included more varied methods of lethal intervention and the landscapes differ (theirs being mountainous and wider while Michigan’s is flatter and surrounded by water on three sides, with attendant differences in vegetation, lake effects, human population density, wolf migration, livestock husbandry practices, etc.). In addition, the survival analyses employed by [4] differed from ours in ways that we could not resolve despite several email exchanges with the lead author and the analyst co-author.

First, [4] did not account for treatment effects beyond a single spatial scale (see Box 1). Their analysis was restricted to the affected wolf pack territory, despite their own reports that killing wolves had at times scattered surviving pack members beyond their original territory [10, 13, 46]. This previous research would argue for an analysis that examined neighboring areas potentially affected by spill-over from scattered survivors.

Second, apparent shortcomings of the statistical modeling in [4] may have affected its results. Their measure of delay to recurrence for full pack removals spans the time from death of the last pack member to the time when a new pack attacked livestock in the same territory. This measure of delay to next depredation artificially inflates effectiveness because it incorporates a potentially long timespan before a new pack establishes, which probably includes many time-consuming events unrelated to the intervention (e.g., immigration, breeding). By contrast, our method censored observations at the end of each year, so subjects were compared on a more-equal footing after intervention. For partial removal and no removal interventions in [4], the territory was still occupied by wolves so delays probably did not include as many time-consuming demographic events (if any). Although we understand that their intent was to analyze if depredations could be delayed for longer by killing entire wolf packs, we would argue that the appropriate control for the evaluation of this intervention would be sites with suitable wolf habitat but without an established pack because of events unrelated to killing wolves, such as recolonization of vacant habitat.

Using a biomedical analogy, [4] identified the hospital bed (the pack territory) as the subject rather than the patient (the wolf pack), regardless if the wolf pack is the same or if it dies and is replaced by a new pack. Researchers continued measuring the delay to the next infection (depredation) in that bed over time, without correcting for the delay to arrival of a new patient to that bed if a previous patient dies. The delay to the next infection once a patient dies is contingent on the arrival of a new patient to that empty bed, which has little to do with the intervention implemented to the bed other than making it available for a new patient (with full pack removal). By contrast, in our study the patient (area) is the only patient, each infection receives a treatment, and delay to next infection is always measured for the same patient with a reset each year.

Third, differences with [4] could also potentially arise from different handling of the proportional hazards (PH) assumption. We evaluated the compliance of our models with the PH assumption through the inclusion of a time-varying covariate (tvc) [31]. A significant tvc affects both our treatment hazard ratios and their significance, (e.g., Table 3). We assume that [4]’s team employed other model diagnostics to evaluate their compliance with the PH assumption, but they did not report such diagnostic tests. Until the summary data are published, we cannot agree with the conclusions in [4].

Finally, some might argue that by defining our subjects as area-years and including the same area over different years we pseudo-replicated non-independent samples. In our dataset, only 16 out of 106 sections had depredation incidents in multiple years. To address that concern, we built an alternative model in which areas were omitted in succeeding years (S5 File). Results for this dataset are consistent with our main results at the section scale (S5 File).

## Conclusions

Lethal interventions by the State of Michigan against wolves in the vicinities of verified livestock losses did not appear to reduce future losses. We view our findings as preliminary pending experiments with stronger inference. Our inferences could not overcome a lack of systematic information on government interventions and no effort to control for their treatments, despite a call for such shortly after the legalization of lethal removal of wolves in 2003 [47]. We detected a potential spill-over of depredations from the farm receiving lethal intervention onto neighboring farms. Given this evidence for interactions in depredations over significant areas, we must look with skepticism upon any previous or future results which analyze the functional effectiveness of lethal control but do not take these spatial relationships into account. Further, given the severe ethical issues involved in implementing harmful or lethal interventions, the lack of effectiveness of these interventions argues for their curtailing in favor of non-lethal alternatives that are effective. In the State of Michigan, there is strong scientific evidence [2] for the effectiveness of at least two non-lethal methods (fladry and livestock guarding dogs; 7–8). No peer-reviewed scientific study has ever shown lethal methods to be effective in Michigan. Indeed, our review of [4] above suggests no study in the USA has yet proven with strong inference that killing wolves is effective in preventing future livestock losses [2, 39–41]. Although it may seem obvious that killing a predator whose jaws are about to lock on a calf should protect the calf, government lethal methods are not implemented in that way. Virtually all are indirect methods such as traps placed far from the depredation site and long after a calf is killed. Therefore, rigorous scientific evaluations are a necessary prerequisite before implementing an intervention, especially given the ethical and legal obligations to balance protection of livestock and wild animals for the broad public interest. The US Endangered Species Act mandates the use of the “best scientific and commercial data available” when making conservation and management decision for listed species.

Following recommendations for ethical wildlife management [48, 49], lethal management should be discontinued, as currently the harm it causes wolves and livestock is not offset by benefits. If lethal methods are still necessary in some situations [48, 49], these should be constantly monitored and evaluated by independent third parties to measure their effectiveness or lack thereof [48].

## Supporting information

S1 File Distribution of observations and recurrent events between treatments and strata for all spatial scales. (DOCX)

S2 File Results from frailty mfodels for main dataset, for all spatial scales. (DOCX)

S3 File Results for ‘traps placed’ dataset. (DOCX)

S4 File Results for post-2003 dataset. (DOCX)

S5 File Results for ‘skip-a-year’ dataset and outlier exclusion. (DOCX)

S1 Data File Livestock depredation events involving gray wolves in the state of Michigan, USA (1998–2014). (XLSX)

## References

[1] Woodroffe R, Redpath SM. When the hunter becomes the hunted. Science. 2015;348(6241):1312–4. doi: 10.1126/science.aaa8465 26089495 10.1126/science.aaa8465

[2] Treves A, Krofel M, McManus J. Predator control should not be a shot in the dark. Front Ecol Environ. 2016;14(7):380–8.

[3] Herfindal I, Linnell JDC, Moa PF, Odden J, Austmo LB, Andersen R. Does recreational hunting of lynx reduce depredation losses of domestic sheep? J Wildl Manage. 2005;69:1034–42.

[4] Bradley EH, Robinson HS, Bangs EE, Kunkel K, Jimenez MD, Gude JA, et al Effects of wolf removal on livestock depredation recurrence and wolf recovery in Montana, Idaho, and Wyoming. The Journal of Wildlife Management. 2015;79(8):1337–46. doi: 10.1002/jwmg.948

[5] Mukherjee S. The Emperor of All Maladies: A Biography of Cancer Mew York: Scribner; 2010.

[6] Platt JR. Strong inference. science. 1964;146(3642):347–53. doi: 10.1126/science.146.3642.347 17739513 10.1126/science.146.3642.347

[7] Davidson-Nelson SJ, Gehring TM. Testing fladry as a nonlethal management tool for wolves and coyotes in Michigan. Human–Wildlife Interactions. 2010;4:87–94.

[8] Gehring TM, VerCauteren KC, Provost ML, Cellar AC. Utility of livestock-protection dogs for deterring wildlife from cattle farms. Wildl Res. 2010;37(8):715–21.

[9] Wielgus RB, Peebles KA. Effects of wolf mortality on livestock depredations. PLoS One. 2014;9(12):e113505 doi: 10.1371/journal.pone.0113505 ; PubMed Central PMCID: PMCPMC4254458. 25470821 10.1371/journal.pone.0113505 PMC4254458

[10] Bradley EH, Pletscher DH. Assessing factors related to wolf depredation of cattle in fenced pastures in Montana and Idaho. Wildl Soc Bull. 2005;33(4):1256–65.

[11] Bradley EH, Pletscher DH, Bangs EE, Kunkel KE, Smith DW, Mack CM, et al Evaluating wolf translocation as a nonlethal method to reduce livestock conflicts in the northwestern United States. Conserv Biol. 2005;19:1498–508.

[12] Poudyal N, Baral N, Asah ST. Wolf Lethal Control and Livestock Depredations: Counter-Evidence from Respecified Models. PLOS ONE. 2016;11(2):e0148743 doi: 10.1371/journal.pone.0148743 26866592 10.1371/journal.pone.0148743 PMC4751083

[13] Bradley EH. Evaluation of wolf-livestock conflicts and management in the northwestern United States: University of Montana; 2004.

[14] Treves A, Martin KA, Wydeven AP, Wiedenhoeft JE. Forecasting Environmental Hazards and the Application of Risk Maps to Predator Attacks on Livestock. Bioscience. 2011;61(6):451–8. doi: 10.1525/bio.2011.61.6.7

[15] Linnell JD, Odden J, Smith ME, Aanes R, Swenson JE. Large carnivores that kill livestock: do" problem individuals" really exist? Wildl Soc Bull. 1999:698–705.

[16] Refsnider RL. The role of the Endangered Species Act in Midwest wolf recovery In: Wydeven AP, Van Deelan TR, Heske E, editors. Recovery of gray wolves in the Great Lakes Region of the United States. New York: Springer; 2009 p. 311–29.

[17] Edge JL, Beyer DE Jr, Belant JL, Jordan MJ, Roell BJ. Adapting a predictive spatial model for wolf Canis spp. predation on livestock in the Upper Peninsula, Michigan, USA. Wildl Biol. 2011;17:1–10.

[18] Edge JL, Beyer DE Jr, Belant JL, Jordan MJ, Roell BJ. Livestock and domestic dog predations by wolves in Michigan. Human-Wildlife Interactions. 2011;5:66–78.

[19] Beyer DE, Peterson R.O., Vucetich J.A., Hammill JH. Wolf population changes in Michigan In: Wydeven AP, Van Deelen TR, Heske EJ, editors. Recovery of Gray Wolves in the Great Lakes Region of the United States: an Endangered Species Success Story. New York: Springer; 2009 p. 65–85.

[20] Ruid DB, Paul WJ, Roell BJ, Wydeven AP, Willging RC, Jurewicz RL, et al Wolf–human conflicts and management in Minnesota, Wisconsin, and Michigan In: Wydeven AP, Van Deelan TR, Heske E, editors. Recovery of gray wolves in the Great Lakes region of the United States. New York: Springer; 2009 p. 279–95.

[21] Olson ER, Stenglein JL, Shelley V, Rissman AR, Browne‐Nuñez C, Voyles Z, et al Pendulum swings in wolf management led to conflict, illegal kills, and a legislated wolf hunt. Conservation Letters. 2014 doi: 10.1111/conl.12096

[22] Treves A, Jurewicz RR, Naughton Treves L, Rose RA, Willging RC, Wydeven AP. Wolf depredation on domestic animals in Wisconsin, 1976–2000. Wildl Soc Bull. 2002;30:231–41.

[23] Wydeven AP, Treves A, Brost B, Wiedenhoeft JE. Characteristics of wolf packs in Wisconsin: identification of traits influencing depredation People and predators: from conflict to coexistence Island Press, Washington, DC 2004:28–50.

[24] Bump JK, Murawski CM, Kartano LM, Beyer DE, Roell BJ. Bear-Baiting May Exacerbate Wolf-Hunting Dog Conflict. PLOS One. 2013; doi: 10.1371/journal.pone.0061708 23613910 10.1371/journal.pone.0061708 PMC3629141

[25] Flesher J. Michigan hunt targeted problem wolves, DNR says Detroit Free Press 2014 26 1 2014.

[26] Musiani M, Visalberghi E. Effectiveness of fladry on wolves in captivity. Wildl Soc Bull. 2001;29:91–8.

[27] Voyles Z, Treves A, MacFarland D. Spatiotemporal effects of nuisance black bear management actions in Wisconsin. Ursus. 2015;26(1):11–20.

[28] MAS S. Michigan Agricultural Statistics. 2012.

[29] Chavez AS, Gese EM. Landscape use and movement of wolves in relation to livestock in a wildland-agriculture matrix. J Wildl Manage. 2006;70(4):1079–86.

[30] Wydeven AP, Wiedenhoeft JE, Schultz RN, Thiel RP, Jurewicz RL, Kohn BE, et al History, population growth, and management of wolves in Wisconsin In: Wydeven AP, Van Deelan TR, Heske E, editors. Recovery of gray wolves in the Great Lakes Region of the United States. New York: Springer; 2009 p. 87–105.

[31] Hosmer DW Jr, Lemeshow S, May S. Applied survival analysis: Regression modelling of time to event data Second Edition Second Edition ed. Hoboken, New Jersey, USA: Wiley-Interscience; 2008.

[32] Clark TG, Bradburn MJ, Love SB, Altman DG. Survival Analysis Part I: Basic concepts and first analyses. Br J Cancer. 2003;89(2):232–8. doi: 10.1038/sj.bjc.6601118 PubMed PMID: PMC2394262. 12865907 10.1038/sj.bjc.6601118 PMC2394262

[33] Lin DY, Wei L-J. The robust inference for the Cox proportional hazards model. Journal of the American statistical Association. 1989;84(408):1074–8.

[34] Chapron G, Treves A. Blood does not buy goodwill: allowing culling increases poaching of a large carnivore. Proceedings of the Royal Society of London B: Biological Sciences. 2016;283(1830). doi: 10.1098/rspb.2015.2939 27170719 10.1098/rspb.2015.2939 PMC4874699

[35] Kelly PJ, Lim LLY. Survival analysis for recurrent event data: an application to childhood infectious diseases. Stat Med. 2000;19(1):13–33. 10623910 10.1002/(sici)1097-0258(20000115)19:1<13::aid-sim279>3.0.co;2-5

[36] Treves A, Bruskotter JT. Gray wolf conservation at a crossroads. Bioscience. 2011;61:584–5.

[37] Xu Y, Cheung YB. Frailty models and frailty-mixture models for recurrent event times. Stata J. 2015;15(1):135–54.

[38] Treves A, Naughton-Treves L, Harper EK, Mladenoff DJ, Rose RA, Sickley TA, et al Predicting human-carnivore conflict: a spatial model derived from 25 years of data on wolf predation on livestock. Conserv Biol. 2004;18(1):114–25.

[39] van Eeden LM, Crowther MS, Dickman CR, Macdonald DW, Ripple WJ, Ritchie EG, et al Managing conflict between large carnivores and livestock. Conserv Biol. 2017:n/a-n/a. doi: 10.1111/cobi.12959 28556528 10.1111/cobi.12959

[40] Miller JRB, Stoner KJ, Cejtin MR, Meyer TK, Middleton AD, Schmitz OJ. Effectiveness of contemporary techniques for reducing livestock depredations by large carnivores. Wildl Soc Bull. 2016:n/a-n/a. doi: 10.1002/wsb.720

[41] Eklund A, López-Bao JV, Tourani M, Chapron G, Frank J. Limited evidence on the effectiveness of interventions to reduce livestock predation by large carnivores. Scientific Reports. 2017;7(1):2097 doi: 10.1038/s41598-017-02323-w 28522834 10.1038/s41598-017-02323-w PMC5437004

[42] Hogberg J, Treves A, Shaw B, Naughton-Treves L. Changes in attitudes toward wolves before and after an inaugural public hunting and trapping season: early evidence from Wisconsin's wolf range. Environ Conserv. 2015:1–11. doi: 10.1017/s037689291500017x

[43] Treves A, Naughton-Treves L, Shelley V. Longitudinal analysis of attitudes toward wolves. Conserv Biol. 2013;27(2):315–23. doi: 10.1111/cobi.12009 PubMed PMID: 23293913. 23293913 10.1111/cobi.12009

[44] Treves A, Vucetich JA, Rabenhorst M, Cornman A. An evaluation of localized wolf control efforts to prevent subsequent livestock depredation in Michigan. Natural Resources Report No 2013–4 Little River Band of Ottawa Indians. 2013.

[45] Haber GC. Biological, conservation, and ethical implications of exploiting and controlling wolves. Conserv Biol. 1996;10:1068–81.

[46] Brainerd SM, Andrén H, Bangs EE, Bradley EH, Fontaine JA, Hall W, et al The effects of breeder loss on wolves. The Journal of Wildlife Management. 2008;72(1):89–98.

[47] Treves A, Naughton-Treves L. Evaluating lethal control in the management of human-wildlife conflict In: Woodroffe GL, Thirgood S, Rabinowitz A, editors. People and Wildlife, Conflict or Coexistence? Cambridge, UK: Cambridge University Press; 2005 p. 86–106.

[48] Lynn WS. Barred owls in the Pacific Northwest: An ethics brief. 2012.

[49] Dubois S, Fenwick N, Ryan EA, Baker L, Baker SE, Beausoleil NJ, et al International consensus principles for ethical wildlife control. Conserv Biol. 2017. 10.1111/cobi.12896 28092422

