# Correction: Killing wolves to prevent predation on livestock may protect one farm but harm neighbors

**DOI:** 10.1371/journal.pone.0209716

**Published:** 2018-12-19

**Authors:** Francisco J. Santiago-Avila, Ari M. Cornman, Adrian Treves

The hazard ratio is incorrect throughout the article.

There is an error in the sixth sentence of the Abstract. The correct sentence is: Hazard ratios suggest lethal intervention was associated with an insignificant 27% lower risk of recurrence of events at trapping sites, but offset by an insignificant 78% increase in risk of recurrence at sites up to 5.42 km distant in the same year, compared to the non-lethal treatment.

There is an error in sixth sentence under the ‘Township Scale’ heading of the Results Section. The correct sentence is: Lethal intervention increased risk (hastening recurrence) by 78%, but this was not statistically significant (treatment HR = 1.78, P = 0.458).

There are errors in the fourth sentence of the Discussion Section. The correct sentence is: A small (27%), statistically insignificant reduction in the risk of depredation at the section level was more than offset by a 2.9 times larger (78%) yet also statistically insignificant increase in the risk of depredation at the township scale which is about half the size of a wolfpack territory, and then another statistically insignificant decrease (28%) in risk at the scale of neighborhood of townships, which is four times larger than the average wolfpack territory [30].
