# Supplementary material for: Killing wolves to prevent predation on livestock may protect one farm but harm neighbors
Source: PLoS One. 2018 Jan 10;13(1):e0189729. doi: 10.1371/journal.pone.0189729 (PMC5761834; doi:10.1371/journal.pone.0189729)
Supplement: S1 File — (DOCX) [file pone.0189729.s001.docx]

**S1 File: Distribution of observations and recurrent events between treatments and strata for all spatial scales**

Section scale: At the section scale, we restricted analyses to 3 strata due to lack of depredation events (n<10) in subsequent strata (Table A).

**Table A.** Number of depredation events per intervention type, by strata (S#)

|  | Number of observations (n) | | | | | | | | | | | |
| --- | --- | --- | --- | --- | --- | --- | --- | --- | --- | --- | --- | --- |
| Intervention / Stratum | S1 | S2 | S3 | S4 | S5 | S6 | S7 | S8 | S9 | S10 | S11 | S12 |
| Lethal | 23 | 3 | 5 | 1 |  |  |  |  |  |  |  |  |
| Non-lethal | 128 | 28 | 12 | 7 | 4 | 3 | 3 | 3 | 3 | 3 | 3 | 1 |

**Table B.** Number of censored (0) and recurrent (1) depredation events for non-lethal treatment, by strata (S#)

|  | Number of observations (n) | | | | | | | | | | | |
| --- | --- | --- | --- | --- | --- | --- | --- | --- | --- | --- | --- | --- |
| Rec event / Stratum | S1 | S2 | S3 | S4 | S5 | S6 | S7 | S8 | S9 | S10 | S11 | S12 |
| 0 | 100 | 12 | 8 | 3 | 1 |  |  |  |  |  | 2 | 1 |
| 1 | 28 | 16 | 4 | 4 | 3 | 3 | 3 | 3 | 3 | 3 | 1 |  |

**Table C.** Number of censored (0) and recurrent (1) depredation events for lethal treatment, by strata (S#)

|  | Number of observations (n) | | | |
| --- | --- | --- | --- | --- |
| Rec event / Stratum | S1 | S2 | S3 | S4 |
| 0 | 20 | 2 | 1 | 1 |
| 1 | 3 | 1 | 4 |  |

Township scale: At the township scale, we restricted analyses to one strata due to lack of depredation events for both treatments in stratum 2 (Tables D-F).

**Table D.** Number of depredation events per type of intervention type, by strata (S#)

|  | Number of observations (n) | | |
| --- | --- | --- | --- |
| Intervention / Stratum | S1 | S2 | S3 |
| Lethal | 26 | 2 |  |
| Non-lethal | 99 | 22 | 3 |

**Table E.** Number of censored (0) and recurrent (1) depredation events for non-lethal treatment, by strata (S#)

|  | Number of observations (n) | | |
| --- | --- | --- | --- |
| Rec event / Stratum | S1 | S2 | S3 |
| 0 | 78 | 19 | 3 |
| 1 | 21 | 3 |  |

**Table F.** Number of censored (0) and recurrent (1) depredation events for lethal treatment, by strata (S#)

|  | Number of observations (n) | |
| --- | --- | --- |
| Rec event / Stratum | S1 | S2 |
| 0 | 23 | 2 |
| 1 | 3 |  |

Neighborhood scale: At the neighborhood scale, we restricted analyses to 2 strata due to lack of depredation events (n<10) in subsequent strata (Table G).

**Table G.** Number of depredation events per type of intervention type, by strata (S#)

|  | Number of observations (n) | | | |
| --- | --- | --- | --- | --- |
| Intervention / Stratum | S1 | S2 | S3 | S4 |
| Lethal | 20 | 6 | 1 |  |
| Non-lethal | 86 | 13 | 5 | 1 |

**Table H.** Number of censored (0) and recurrent (1) depredation events for non-lethal treatment, by strata (S#)

|  | Number of observations (n) | | | |
| --- | --- | --- | --- | --- |
| Rec event / Stratum | S1 | S2 | S3 | S4 |
| 0 | 72 | 8 | 4 | 1 |
| 1 | 15 | 5 | 1 |  |

**Table I.** Number of censored (0) and recurrent (1) depredation events for lethal treatment, by strata (S#)

|  | Number of observations (n) | | |
| --- | --- | --- | --- |
| Rec event / Stratum | S1 | S2 | S3 |
| 0 | 15 | 5 | 1 |
| 1 | 4 | 1 |  |
