# Supplementary material for: Killing wolves to prevent predation on livestock may protect one farm but harm neighbors
Source: PLoS One. 2018 Jan 10;13(1):e0189729. doi: 10.1371/journal.pone.0189729 (PMC5761834; doi:10.1371/journal.pone.0189729)
Supplement: S2 File — (DOCX) [file pone.0189729.s002.docx]

**S2 File: Results from frailty models, for all spatial scales**

Frailty models evaluate the goodness of fit of the treatment variable including within-subject random effects that might confound the effect of treatment [35]. In our context, frailty models assume that there are high-risk and low-risk subjects due to factors extrinsic to treatments or individual depredation events [17, 36]. Frailty models are useful when survival time is influenced by unmeasured factors [31, 37]. Important differences between subjects can confound the apparent effect of treatment [35]. Our frailty model results supported our main models, showing an insignificant reduction in risk of recurrence following lethal intervention, at all scales. Only the section scale frailty model revealed high heterogeneity due to within-subject effects (Table A). The section scale of our models is precisely the level at which one would expect the most frailty (within-subject factors would be felt at the spatial scale closest to an individual farm). For example, two farms with unusually high numbers of depredations (see S5 File) might have been detected by our frailty model. Accounting for this heterogeneity thus increased the magnitude of the coefficient for the effect of the intervention (HR = 52%) relative to our main model, although the coefficient for lethal intervention remains statistically insignificant.

**Table A**. Results from frailty models measuring risk of recurrence between treatments (lethal and non-lethal) implemented after depredation events, for all spatial scale

|  | **Spatial scale of analysis** | | |
| --- | --- | --- | --- |
|  | **Section** | **Township** | **Neighborhood** |
| **Frailty models** |  |  |  |
| *Intervention COEF (SD)* | -0.74 (0.522) | -0.73 (0.620) | -0.22 (0.510) |
| p-val | 0.158 | 0.242 | 0.67 |
| *frailty COEF (SD)* | 4.65 (1.68)** | 0.05 | 0.03 |
| p-val | 0.006 | - | - |

*Significance: * if p-val <.05; ** if <.01.*
