# Supplementary material for: Killing wolves to prevent predation on livestock may protect one farm but harm neighbors
Source: PLoS One. 2018 Jan 10;13(1):e0189729. doi: 10.1371/journal.pone.0189729 (PMC5761834; doi:10.1371/journal.pone.0189729)
Supplement: S3 File — (DOCX) [file pone.0189729.s003.docx]

**S3 File: Results for ‘traps placed’ dataset**

In the alternate ‘traps placed’ model, non-lethal management with zero wolves killed was reclassified as ‘lethal’ because the infrastructure of killing wolves and attendant human influences on the habitat were treated as similar to lethal intervention in which wolves died.

Log rank tests could not distinguish the survival functions between treatments at any spatial scale (all tests P>0.05, Table A). All Cox models also echo our main results, suggesting a statistically insignificant effect of lethal intervention relative to no intervention (Table B). Our most robust models at each scale suggest that lethal intervention was associated with a statistically insignificant reduction in risk of recurrence compared to no intervention at the section scale (treatment HR=0.76, P=0.435); a non-significant increase in risk of recurrence at the township scale (although the hazard ratio increases), signaling a greater risk of recurrence (treatment HR=2.35, P=0.247; tvc HR=0.96, P=0.006); and a non-significant reduction in risk of recurrence at the neighborhood of townships scale (treatment HR=0.64, P=0.327). The risk of recurrence also seemed to increase with calendar-year at all spatial scales (Table B), but this effect was not statistically significant at the township scale (P=0.153). Also, consistent with our main results, we found no evidence of a correlation between delay to recurrence and the number of wolves killed at any spatial scale for those depredation events followed by lethal intervention (Spearman’s rho P>.05; Table C).

**Table A**. General and stratified log-rank (χ^2^) tests examining difference between treatments’ (lethal and non-lethal) survival distributions (measuring risk of recurrence) after wolf depredations, for all spatial scales, for the ‘traps placed’ dataset.

|  | **Spatial scale of analysis** | | |
| --- | --- | --- | --- |
|  | **Section** | **Township** | **Neighborhood** |
| ***INCIDENTS AND 'FAILURES'*** |  |  |  |
| **TOTAL DEPREDATION EVENTS** | 199 | 125 | 125 |
| Failures (recurrent events) | 56 | 24 | 25 |
| ***SURVIVAL FUNCTIONS*** |  |  |  |
| **Log rank test (χ2)** | 0.44 | 0.86 | 0.40 |
| p-val | 0.5072 | 0.3534 | 0.5296 |
| **Stratified Log-rank test (χ2)** | 0.46 | - | 0.68 |
| p-val | 0.4989 | - | 0.4094 |

*Significance: * if p-val <.05; ** if <.01.*

**Table B**. Main results of Cox models measuring risk of recurrence between treatments (lethal and non-lethal) implemented after wolf depredations, for all spatial scales, for the ‘traps placed’ dataset.

|  | **Spatial scale of analysis** | | | | | |
| --- | --- | --- | --- | --- | --- | --- |
|  | **Section** | | **Township** | | **Neighborhood** | |
| ***PROPORTIONAL HAZARD MODELS*** | *Interv* | *Interv & year* | *Interv* | *Interv & year* | *Interv* | *Interv & year* |
| **Standard cox (stratified)** |  |  |  |  |  |  |
| *Intervention HR (SD)* | 0.78 (0.26) | 0.76 (0.27) | 0.60 (0.35) | 0.58 (0.33) | 0.69 (0.32) | 0.64 (0.29) |
| p-val | 0.453 | 0.435 | 0.38 | 0.34 | 0.430 | 0.327 |
| *year HR (SD)* | - | 1.09 (0.04)* | - | 1.05 (0.05) | - | 1.14 (0.06)* |
| p-val | - | 0.022 | - | 0.292 | - | 0.022 |
| **Standard cox with tvc (stratified)** |  |  |  |  |  |  |
| *Intervention HR (SD)* | 0.55 (0.25) | 0.54 (0.26) | 3.46 (2.82) | 3.31 (2.70) | 0.78 (0.57) | 0.75 (0.58) |
| p-val | 0.19 | 0.191 | 0.128 | 0.141 | 0.737 | 0.711 |
| *tvc(Intervention)* | 1.01 (0.01) | 1.01 (0.01) | 0.95 (0.02)** | 0.95 (0.02)** | 1.00 (0.01) | 1.00 (0.01) |
| p-val | 0.129 | 0.15 | 0.006 | 0.006 | 0.821 | 0.769 |
| *year HR (SD)* | - | 1.09 (0.04)* | - | 1.05 (0.05) | - | 1.14 (0.06)* |
| p-val | - | 0.023 | - | 0.291 | - | 0.022 |

*Significance: * if p-val <.05; ** if <.01.*

**Table C**. Spearman correlation between delay to recurrence and number of wolves killed after depredation events followed by lethal intervention (wolves killed > 0), for all spatial scales, for the ‘traps placed’ dataset.

|  | **Section** | **Township** | **Neighborhood** |
| --- | --- | --- | --- |
| Spearman's rho | 0.076 | 0.233 | 0.161 |
| p-val | 0.6571 | 0.224 | 0.3954 |

*Significance: * if p-val <.05; ** if <.01.*
