# Supplementary material for: Killing wolves to prevent predation on livestock may protect one farm but harm neighbors
Source: PLoS One. 2018 Jan 10;13(1):e0189729. doi: 10.1371/journal.pone.0189729 (PMC5761834; doi:10.1371/journal.pone.0189729)
Supplement: S5 File — (DOCX) [file pone.0189729.s005.docx]

**S5 File: Results for ‘skip-a-year’ dataset and outlier exclusion**

Below we present results for our alternate ‘skip-a-year’ model for the section scale, created to address potential pseudo-replication concerns. In this model, sections are left outside the study for one year if they experienced depredations during the previous year. A one-year period outside the study seems a sensible amount of time for the section to enter the study as a different subject (section-year combination) independent from its prior inclusion..

Consistent with our main and supplementary analyses, log rank tests could not distinguish the survival functions between treatments at the section scale (df=1, general test: χ^2^=0.83, P=0.363; stratified test: χ^2^=1.27, P=0.488; Table A). Results for our Cox models are also consistent with our main results, suggesting a statistically insignificant effect of lethal intervention relative to non-lethal intervention (Table B). The models including tvc indicate that violating the proportional hazards assumption is not a concern (tvc P>0.05). Our main model, including treatment and year, shows that lethal intervention was associated with a statistically insignificant 45% reduction in risk of recurrence compared to non-lethal intervention at the section scale (treatment HR=0.52, P=0.145). The risk of recurrence also seemed to increase with calendar-year (HR=1.07; P=0.104), but this effect was not statistically significant. Also consistent with our main results, we found no evidence of a correlation between delay to recurrence and the number of wolves killed for those depredation events followed by lethal intervention (Spearman’s rho P>0.05; Table C).

Additionally, we ran our tests on the dataset excluding one outlier farm which had an atypically high number of depredations (see <http://www.mlive.com/news/index.ssf/2013/11/john_koski_part_1_tour_the_far.html>). Although this restricted the analysis to one stratum at each spatial scale, the results for both section and township echo our main results. Furthermore, the result for increased risk for the township scale became stronger (p=0.093) in this model.

**Table A**. General and stratified log-rank (χ^2^) tests examining difference between treatments’ survival distributions at the section level for the ‘skip-a-year’ dataset.

|  |  |  |
| --- | --- | --- |
|  | **Section** | |
| ***SUBJECTS AND 'FAILURES'*** |  | |
| **TOTAL DEPREDATION EVENTS** | 166 | |
| Failures (recurrent events) | 38 | |
| ***SURVIVAL FUNCTIONS*** |  | |
| **Log rank test (χ2)** | 0.83 | |
| p-val | 0.3633 | |
| **Stratified Log-rank test (χ2)** | 1.27 | |
| p-val | 0.2602 | |

*Significance: * if p-val <.05; ** if <.01.*

**Table B**. Results of Cox models at the section level for the ‘skip-a-year’ dataset.

|  |  |  |
| --- | --- | --- |
|  | **Section** | |
| ***PROPORTIONAL HAZARD MODELS*** | *Interv* | *Interv & year* |
| **Standard cox (stratified)** |  |  |
| *Intervention HR (SD)* | 0.55 (0.24) | 0.52 (0.234) |
| p-val | 0.166 | 0.145 |
| *year HR (SD)* | - | 1.07 (0.043) |
| p-val | - | 0.104 |
| **Standard cox with tvc (stratified)** |  |  |
| *Intervention HR (SD)* | 0.51 (0.24) | 0.48 (0.229) |
| p-val | 0.149 | 0.125 |
| *tvc(Intervention) HR (SD)* | 1.00 (0.009) | 1.00 (0.009) |
| p-val | 0.771 | 0.826 |
| *year HR (SD)* | - | 1.07 (0.043) |
| p-val | - | 0.105 |

*Significance: * if p-val <.05; ** if <.01.*

**Table C**. Spearman correlation between delay to recurrence and number of wolves killed for depredation events followed by lethal intervention (wolves killed > 0) at the section level for the ‘skip-a-year’ dataset.

|  | **Section** |
| --- | --- |
| Spearman's rho | 0.193 |
| p-val | 0.3547 |

*Significance: * if p-val <.05; ** if <.01.*
